# A case of late‐onset psychosis secondary to traumatic brain injury difficult to differentiate from delirium

**DOI:** 10.1002/pcn5.70125

**Published:** 2025-05-20

**Authors:** Tomonobu Kato, Takashi Tsujii, Hisashi Kida, Daiki Mochizuki, Shigeru Nikkuni, Kei Sakuma

**Affiliations:** ^1^ Department of Neuropsychiatry Asaka Hospital Koriyama Fukushima Japan; ^2^ Department of Neuropsychiatry Keio University School of Medicine Shinjuku Tokyo Japan; ^3^ Department of Psychiatry Tokyo Adachi Hospital Adachi Tokyo Japan

**Keywords:** delirium, psychiatric disorder, psychosis secondary to traumatic brain injury, seizure, traumatic brain injury

## Abstract

**Background:**

Traumatic brain injury (TBI) is a brain injury caused by external forces and has a prevalence of 432 cases (95% uncertainty interval [UI]: 413–452) per 100,000 people in Japan. Previous studies have indicated that TBI is associated with numerous psychiatric disorders, including psychosis.

**Case Presentation:**

A 70‐year‐old male with a history of diabetes, bipolar disorder, and TBI sustained at the age of 51 years presented with late‐onset psychotic symptoms. This case highlights the delirium‐like fluctuating nature of psychotic symptoms and their successful management with risperidone.

**Conclusion:**

Late‐onset psychotic disorders due to TBI can emerge long after initial injury and require careful and continuous monitoring.

## Graphical abstract

Traumatic brain injury (TBI) causes psychotic disorders to occur in a delayed‐onset form in certain cases. In this report, we present a case of psychotic disorders due to TBI with a sudden delirium‐like onset nearly 20 years after the traumatic injury, posing significant challenges for differential diagnosis.

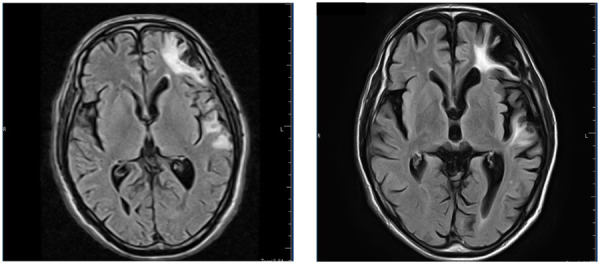

## BACKGROUND

Traumatic brain injury (TBI) is an injury to the brain caused by an external force. Its prevalence is estimated at 599 (95% uncertainty interval [UI]: 573–627) cases per 100,000 people worldwide and 432 (95% UI: 413–452) in Japan.^1^ It results in various neurobehavioral sequelae, such as memory deficits, aggression, apathy, headaches, and seizures.^2^ It is also associated with various psychiatric conditions. The reported prevalence of schizophrenia‐like psychosis following TBI ranges from 1% to 9%,^3^, ^4^ while mood disorders occur in 6%–77% of cases, anxiety disorders in 11%–70%, and challenging behaviors in 11%–90%.^3^, ^4^, ^5^, ^6^ The onset of psychosis after TBI follows a bimodal distribution and can be classified as early‐onset and late‐onset types. A case series reported that 38% of patients developed psychosis within the first year after sustaining a TBI (early onset), while 36% experienced psychosis after 5 years (late onset), with a mean latency of 3.6 years. The sample consisted of 56 men and eight women.^6^, ^7^ Late‐onset psychotic disorders due to TBI (PDTBI) often occur in older adults.^7^ However, in general, late‐onset psychosis is associated with a higher risk of adverse drug reactions compared to early‐onset psychosis.^8^ Additionally, it is often misdiagnosed as schizophrenia^7^ despite having distinct symptomatic features, such as a lower likelihood of negative symptoms and a higher likelihood of positive findings on computed tomography, MRI, or electroencephalography (EEG).^3^ Therefore, accurate diagnosis and appropriate management are essential.

Here, we present a case of late‐onset PDTBI that initially manifested as delirium. The patient presented with psychotic symptoms at night, which worsened over a few days and disturbed the sense of time. Memory of the symptoms was preserved, and the patient was diagnosed with late‐onset PDTBI but not delirium.

## CASE PRESENTATION

**Figure 1:**
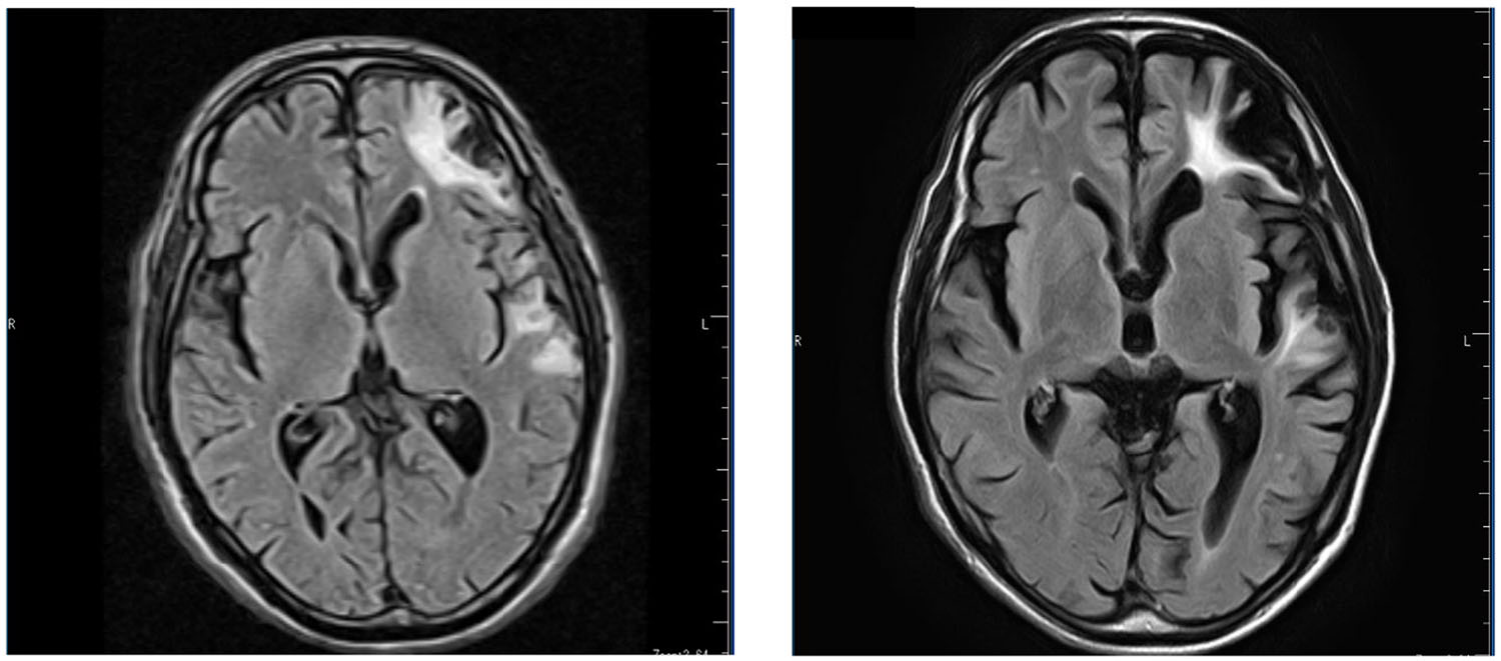
MRI FLAIR images of the patient before and after hospitalization. The left panel shows images at 1 year after the injury, and the right panel shows images on the day before hospitalization.

The patient was a 70‐year‐old male patient with well‐controlled diabetes and bipolar disorder. He also had a family history of bipolar disorder, and his older brother was affected. At the age of 51, he had sustained a skull fracture, resulting in TBI in the left frontal and temporal lobes (Figure 1, left). Since then, he has experienced recurrent epileptic seizures, although the last recorded seizure occurred at the age of 53 years. Cognitive impairment, likely related to trauma, was noted, with a Mini‐Mental State Examination (MMSE) score of 21 at 60 years of age. However, there was no significant progression, with an MMSE score of 22 at 4 months before hospitalization. One month before hospitalization, the patient experienced mood depression. Around the same time, he contracted COVID‐19, leading to frequent absences from daycare services. One week prior to hospitalization, the patient exhibited agitation and wandering at night. He expressed delusional statements, such as “Everyone is dead, gone, and the times feel different,” prompting his hospitalization. This was the first time that his next of kin confirmed psychotic symptoms. However, the following day, he remembered that his family had taken him to the hospital the previous night and he also remembered what he had said. Date and location orientation were sometimes retained; however, when the MMSE was used, they were not retained and were scored as 9. Head MRI showed no new hemorrhage or infarction (Figure 1, right), and EEG was normal.

Although the symptoms fluctuated, the patient often became agitated, which triggered auditory hallucinations. After the symptoms subsided, he reflected on his experiences. He had been taking 24 mg of perospirone hydrochloride hydrate (perospirone) for over a year before hospitalization. Given its prolonged use, we hypothesized that it would no longer be effective. Additionally, previous studies suggest that the effectiveness of perospirone is inferior compared to other second‐generation antipsychotics.^9^ Therefore, risperidone was initiated after hospitalization, and gradually increased from 0.5 to 2 mg over 2.5 weeks, maintaining this dose until discharge (1 month). Upon admission, we considered the possibility of drug‐induced delirium and discontinued medications deemed unnecessary (brotizolam, trihexyphenidyl hydrochloride, furosemide, and lansoprazole). His symptoms resolved within 1 month. One week after symptom resolution, perospirone was gradually tapered and discontinued over 2 weeks, with no symptom recurrence observed for at least 3 months. Upon symptom resolution, the patient's MMSE score was 26.

Prescriptions at the time of admission were as follows: perospirone hydrochloride hydrate, 24 mg; zonisamide, 300 mg; phenytoin, 100 mg; sodium valproate, 1000 mg; brotizolam 0.25 mg; lemborexant 7.5 mg; trihexyphenidyl hydrochloride, 6 mg; miglitol, 150 mg; magnesium oxide, 1320 mg; furosemide, 20 mg; and lansoprazole, 15 mg.

The final discharge prescriptions were risperidone (2 mg), phenytoin (100 mg), sodium valproate (1000 mg), miglitol (150 mg), and magnesium oxide (1320 mg).

## DISCUSSION

This patient exhibited disturbances in attention and awareness that developed over a short period and fluctuated in severity throughout the day. However, it did not disturb cognition; therefore, it did not meet the criteria for delirium.^10^ In addition, the diurnal variation in symptoms suggests that, if considered part of a manic or depressive episode associated with bipolar disorder, the duration was too short to fulfill the diagnostic criteria.^11^ The patient also experienced hallucinations; however, his delusional statements did not meet the definition of delusions, as they lacked sufficient conviction.^12^ Furthermore, he did not exhibit disorganized speech, grossly disorganized or catatonic behavior, or negative symptoms. Therefore, it did not meet the criteria for schizophrenia.^12^ This represents an incomplete combination of symptoms.

Most psychotic symptoms of PDTBI involve delusions (92%) and hallucinations (87%), with persecutory delusions (77%) and auditory hallucinations (92%) being the most common.^7^ In this case, the patient reported auditory hallucinations, such as “run away,” and exhibited with preparing to escape. However, the symptoms subsided with a gradual increase in risperidone. According to previous case series, antipsychotic medication was highly effective in nearly 90% of cases, whereas other patients responded well to antiepileptic drugs, antidepressants, and lithium.^6^, ^7^ Additionally, a previous study reported that the onset age of PDTBI is 33.4 ± 15.4 years, with a delay of onset of 4.1 ± 6.6 years.^3^ Based on these findings, the present patient deviated significantly from these distributions and initially appeared to have delirium, making it difficult to suspect TBI as the underlying cause. However, the patient's clinical course followed the typical symptomatic and therapeutic course of PDTBI. Previous studies have shown that TBI can lead to the long‐term development of tau pathology in both humans and animal models.^13^, ^14^ This is one possible mechanism for late‐onset PDTBI. Unlike Alzheimer's disease, this process is thought to involve distinct tau lesions.^15^ In this case, the patient's cognitive impairment compared to his pre‐hospitalization state was temporary and reversible, and he was not diagnosed with dementia, which is consistent with this neuropathology.

A differential diagnosis that could not be completely ruled out in this case was drug‐induced psychosis. The patient had been taking 300 mg of zonisamide, which causes psychosis,^16^ daily for 12 years as part of the epilepsy treatment. The disappearance of auditory hallucinations occurred approximately 1 week after increasing risperidone to 2 mg and discontinuing zonisamide, making it difficult to determine which intervention was responsible. However, the zonisamide serum concentration at hospitalization was 4.7 μg/mL, well below the therapeutic range, making this possibility unlikely. Additionally, several medications (brotizolam, trihexyphenidyl hydrochloride, furosemide, and lansoprazole) were discontinued upon admission. Considering that it took approximately 1 month for the symptoms to resolve, it is unlikely that these medications were the primary cause of the psychotic symptoms. Nevertheless, the possibility of a multidrug potentiation effect cannot be completely ruled out. Similarly, we cannot completely rule out the effects of prior COVID‐19 infection, as previous studies have reported new‐onset psychotic disorder in 0.42% of cases following COVID‐19 infection^17^; therefore, it was extremely unlikely, but could be a risk factor.

## CONCLUSION

Late‐onset PDTBI can occur long after initial injury, necessitating careful and continuous monitoring. For patients with TBI‐associated epilepsy, it is advisable to use medications that are less likely to induce psychotic symptoms as side‐effects.

## AUTHOR CONTRIBUTION

Tomonobu Kato and Takashi Tsujii treated the patient at the hospital, whereas Daiki Mochizuki and Shigeru Nikkuni treated the patient at an outpatient clinic. Tomonobu Kato wrote the manuscript. All authors participated in the discussion, writing, and revisions and read and approved the final version of the manuscript.

## CONFLICT OF INTEREST STATEMENT

The authors declare no conflict of interest.

## ETHICS APPROVAL STATEMENT

N/A.

## PATIENT CONSENT STATEMENT

The patient provided verbal informed consent and records were made regarding the method and content of the explanation as well as the details of the consent obtained.

## CLINICAL TRIAL REGISTRATION

N/A.

## Data Availability

N/A.
